# Induction of Protective Immunity to Cryptococcal Infection in Mice by a Heat-Killed, Chitosan-Deficient Strain of *Cryptococcus neoformans*

**DOI:** 10.1128/mBio.00547-16

**Published:** 2016-05-10

**Authors:** Rajendra Upadhya, Woei C. Lam, Brian Maybruck, Charles A. Specht, Stuart M. Levitz, Jennifer K. Lodge

**Affiliations:** a Department of Molecular Microbiology, Washington University School of Medicine, St. Louis, Missouri, USA; b Department of Medicine, University of Massachusetts Medical School, Worcester, Massachusetts, USA

## Abstract

*Cryptococcus neoformans* is a major opportunistic fungal pathogen that causes fatal meningoencephalitis in immunocompromised individuals and is responsible for a large proportion of AIDS-related deaths. The fungal cell wall is an essential organelle which undergoes constant modification during various stages of growth and is critical for fungal pathogenesis. One critical component of the fungal cell wall is chitin, which in *C. neoformans* is predominantly deacetylated to chitosan. We previously reported that three chitin deacetylase (CDA) genes have to be deleted to generate a chitosan-deficient *C. neoformans* strain. This *cda1Δ2Δ3Δ* strain was avirulent in mice, as it was rapidly cleared from the lungs of infected mice. Here, we report that clearance of the *cda1Δ2Δ3Δ* strain was associated with sharply spiked concentrations of proinflammatory molecules that are known to be critical mediators of the orchestration of a protective Th1-type adaptive immune response. This was followed by the selective enrichment of the Th1-type T cell population in the *cda1Δ2Δ3Δ* strain-infected mouse lung. Importantly, this response resulted in the development of robust protective immunity to a subsequent lethal challenge with a virulent wild-type *C. neoformans* strain. Moreover, protective immunity was also induced in mice vaccinated with heat-killed *cda1Δ2Δ3Δ* cells and was effective in multiple mouse strains. The results presented here provide a strong framework to develop the *cda1Δ2Δ3Δ* strain as a potential vaccine candidate for *C. neoformans* infection.

## IMPORTANCE

The most commonly used anticryptococcal therapies include amphotericin B, 5-fluorocytosine, and fluconazole alone or in combination. Major drawbacks of these treatment options are their limited efficacy, poor availability in limited resource areas, and potential toxicity. The development of antifungal vaccines and immune-based therapeutic interventions is promising and an attractive alternative to chemotherapeutics. Currently, there are no fungal vaccines in clinical use. This is the first report of a *C. neoformans* deletion strain with an avirulent phenotype in mice exhibiting protective immunity when used as a vaccine after heat inactivation, although other strains that overexpress fungal or murine proteins have recently been shown to induce a protective response. The data presented here demonstrate the potential for developing the avirulent *cda1Δ2Δ3Δ* strain into a vaccine-based therapy to treat *C. neoformans* infection.

## INTRODUCTION

Cryptococcal meningitis is the most frequent result of *Cryptococcus neoformans* infection of the central nervous system, observed mainly in patients with AIDS. Worldwide, it has been estimated that cryptococcal meningitis accounts for more than 1 million cases with about 625,000 deaths annually (1). Even though infections due to *C. neoformans* are more common, *Cryptococcus gattii* is emerging as an important fungal pathogen with significant virulence, wide-spread environmental prevalence, and the ability to cause infections even in immunocompetent individuals (2, 3). The anticryptococcal treatment regimen of choice consists of a combination of amphotericin B and 5-fluorocytosine. Unfortunately, this combination can have substantial toxicity and is not available in much of the developing world, where most cases are seen (4, 5). In some regions, fluconazole is widely used as an alternate to amphotericin B. However, it is not as effective, and there are reports of strains of *C. neoformans* that have developed resistance to these drugs (6). Although echinocandins are effective for treating other fungal infections, they are ineffective against *C. neoformans* infections. Therefore, there is an urgent need for the development of safe and effective treatment strategies against cryptococcal infections. The development of vaccine-based immunotherapeutics is an attractive alternative for controlling cryptococcal infections.

Upon entering the host, *Cryptococcus* is initially challenged by the complement system and the phagocytic activity of different innate immune cells. Innate defense is specifically triggered by the recognition of the pathogen by pattern recognition receptors (PRRs) on the surface of immune cells. *C. neoformans* is able to modulate host immune responses through a combination of its polysaccharide capsule- and cell wall-associated mannans, mannoproteins, glucans, and chitin. The adaptive immune response against *Cryptococcus* includes both antibody- and cell-mediated responses. Effective cross talk between the innate and adaptive arms of the immune system is critical for the defense against the pathogen and the resolution of the fungal infection (7–9). It is well established that cell-mediated immunity (CMI) plays a critical role in anticryptococcal defense, as is evident from the higher prevalence of cryptococcal infections in immunocompromised patients (1). This is recapitulated in animal models of cryptococcosis, where either immunodeficient transgenic mice or mice that are depleted of CD4^+^ and/or CD8^+^ T cells succumb to cryptococcal infection more rapidly than immunocompetent mice (10, 11). To further support the importance of an adaptive response, several studies have demonstrated a role for humoral immunity in contributing to host protection against experimental cryptococcal infections (12). Antigens demonstrated to induce partial protective immunity include glucuronoxylomannan (GXM), which is a component of the cryptococcal capsule, peptide mimotopes of GXM, complex mixtures of cell surface mannoproteins, and melanin (13–16). Moreover, fungal β-glucan particles have been exploited as an adjuvant and, also, as a vaccine delivery system due to their ability to stimulate dendritic cells to secrete cytokines that mediate beneficial host immune responses and to cause robust stimulation of T cells in murine vaccine models (17, 18).

Evidence that a strain of *C. neoformans* could confer complete protective immunity came from studies of a *C. neoformans* H99 strain that was engineered to express and secrete murine gamma interferon (IFN-γ). In this case, a protective response was mediated primarily by a Th1-type T cell immune response, without the contribution of a B-cell mediated processes (19–21). Vaccination of mice with IFN-γ-expressing *C. neoformans* (strain H99γ) resulted in significant increases in the levels of Th1-type proinflammatory cytokines and chemokines in the lung, with concomitant decreases in the levels of cytokines that are mediators of Th2-type anti-inflammatory activities. This polarized Th1 immunogenic response was associated with clearance of the H99γ cells used for vaccination and, in turn, induced protective immunity against a lethal pulmonary infection with wild-type *C. neoformans* (19). While the H99γ strain described in the above-mentioned study exploited an inflammatory cytokine of the host to induce protective immunity, a strain of *C. neoformans* in which the *SLG1* gene was deleted was recently reported to induce protective immunity in mice both under immunocompetent and experimentally induced immunocompromised conditions (22). *SGL1* encodes a sterylglucosidase that when deleted results in the accumulation of sterylglucosides, which in turn modulates immune responses (23). Therefore, protective immunity developed during infection with an *sgl1*Δ strain is shown to be triggered by the *C. neoformans*-derived sterylglucosides (22).

The cryptococcal cell wall is essential for survival, and its components are critical for mediating host-pathogen interactions. The modified fungal cell wall architecture in a mutant strain may provoke a different host immune response than its wild-type counterpart during infection. This was recently demonstrated by a *C. neoformans* strain engineered to overexpress Znf2, a master regulator of hyphal development. The A/J mouse strain vaccinated with the Znf2-overexpressing strain showed an altered host immune response that resulted in the induction of protective immunity, even when the strain was heat killed (HK) (24).

The *C. neoformans* cell wall differs from those of other pathogenic fungi by the presence of a polysaccharide capsule. In addition, we have previously discovered that, unlike other yeasts, the chitin fiber in the cell wall of *C. neoformans* predominantly exists in its deacetylated chitosan form under laboratory growth conditions and in the infected mouse lung (25). We found that deletion of three *C. neoformans* chitin deacetylase genes, *CDA1*, *CDA2*, and *CDA3*, was sufficient to render the yeast cells devoid of chitosan. The *cda1Δ2Δ3Δ* strain was avirulent in an inhalation infection model in mice and was rapidly cleared from the lungs of infected mice (25). The inability of the mutant yeast cells to deacetylate chitin results in compromised cell wall integrity, as revealed by their increased sensitivity to cell wall stressors and altered melanin phenotype (26). Therefore, we hypothesized that chitosan-deficient yeast cells trigger an altered host immune response compared to the host response to the wild type, which in turn is responsible for their efficient eradication from the host lung. In the present study, we extend our observation of rapid mutant fungal clearance and show that the clearance of the chitosan-deficient *cda1Δ2Δ3Δ* strain was associated with a strong induction of the proinflammatory immune response which was able to specifically attract Th1-type adaptive T cells to the site of infection. We further demonstrate that pulmonary vaccination with the chitosan-deficient strain was able to stimulate a robust protective immunity to a subsequent lethal challenge with a virulent wild-type *C. neoformans* strain in multiple mouse strains. We also report here that heat-killed cells of the *cda1Δ2Δ3Δ* strain were able to induce effective protective immunity to a pulmonary challenge with wild-type *C. neoformans*. The observation that viability of the mutant strain is not required to elicit protective immunity indicates a means to an even-safer vaccine.

## RESULTS

### Chitosan-deficient *cda1Δ2Δ3Δ C. neoformans* strain was rapidly cleared from the infected mouse lung.

**FIG 1:**
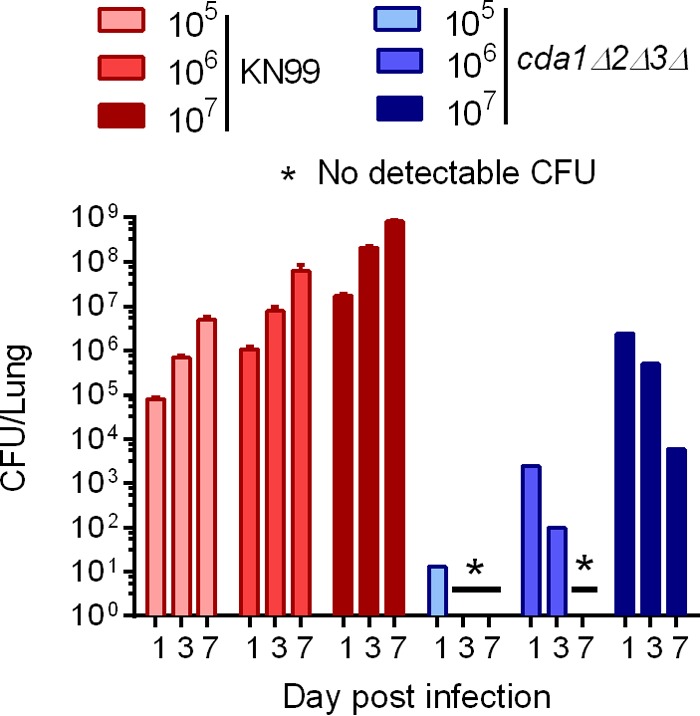
The time required for the host to completely eradicate chitosan-deficient *cda1Δ2Δ3Δ* cells in the lung depends on the size of the yeast inoculum. CBA/J mice were inoculated with various doses (equivalent to 10^5^, 10^6^, or 10^7^ CFU) of either strain KN99α or *cda1Δ2Δ3Δ* mutant cells through intranasal inoculation. At 1, 3, and 7 days p.i., lungs were harvested, homogenized, and serially diluted for CFU enumeration by plating on YPD. Fungal burden is expressed as CFU per lung. The detection limit of the assay was <10 CFU/lung. Error bars indicate standard errors of the means for three mice per treatment group.

We previously reported that the chitosan-deficient *cda1Δ2Δ3Δ* strain was avirulent in CBA/J mice when an inhalation mode of infection was used. This avirulent phenotype was associated with the rapid clearance of the mutant strain from the infected lung when 10^5^ yeast cells were used for infection (25). To characterize the host factors potentially responsible for rapidly clearing the *cda1Δ2Δ3Δ* mutant, we first determined whether the ability of the host to eradicate fungal cells depends on the number of yeast cells used for inoculation. Therefore, we infected CBA/J mice with 10^5^, 10^6^, and 10^7^ CFU of the wild-type, KN99α, and *cda1*Δ2Δ3Δ mutant strains. At 1, 3, and 7 days postinfection (p.i.), lungs were excised from the infected mice and the fungal burdens were determined. Mice infected with 10^5^ CFU of the *cda1Δ2Δ3Δ* strain completely eliminated live fungal cells by day 3 p.i. (Fig. 1). Mice inoculated with 10^6^ CFU of the *cda1Δ2Δ3Δ* mutant had cleared more than 99% of the initial inoculum by day 1 p.i., with complete removal by day 7 p.i. (Fig. 1). Mice infected with 10^7^ CFU of the *cda1Δ2Δ3Δ* mutant, however, took significantly longer to clear the yeast cells. At day 1 p.i., 2 × 10^6^ CFU were recovered, and at day 7 p.i., there were still ~5,000 CFU recovered in the lungs (Fig. 1). In a separate experiment, we performed fungal burden analysis beyond 7 days p.i. for the 10^7^ CFU-infected group and found no detectable CFU on day 10 p.i., indicating that all live chitosan-deficient cells were completely eliminated from the host lung (data not shown). The fungal burden in the lungs of the mice infected with the wild-type KN99α strain continued to proliferate as expected (Fig. 1).

### Clearance of the *cda1Δ2Δ 3Δ* mutant is associated with robust stimulation of cytokines that are critical for inducing protective immunity.

**FIG 2:**
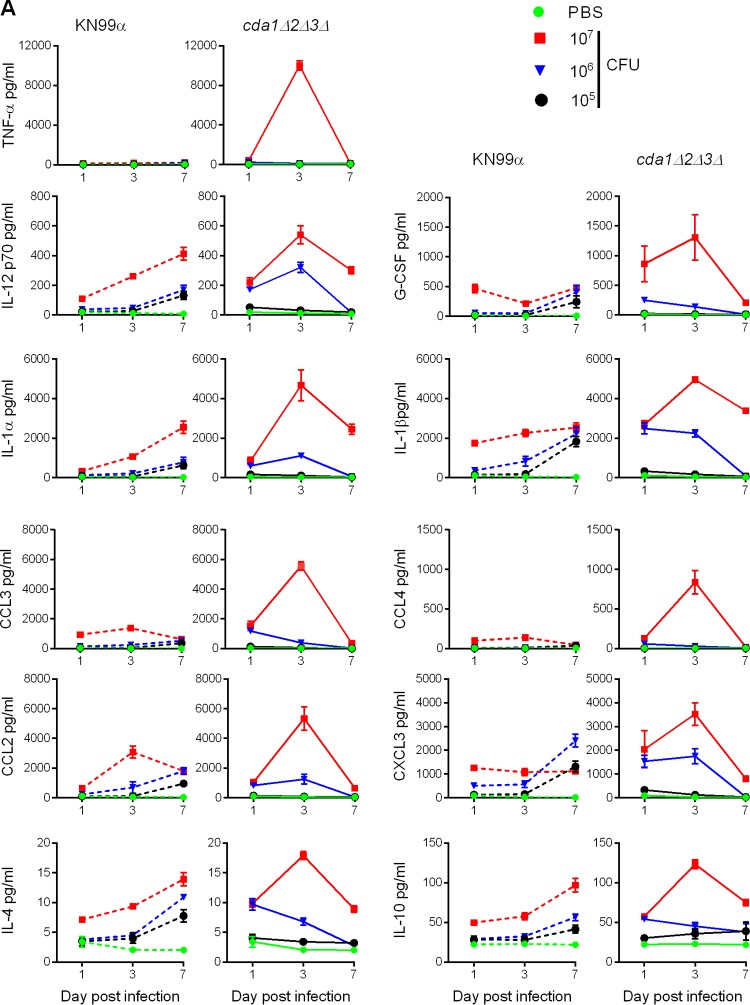

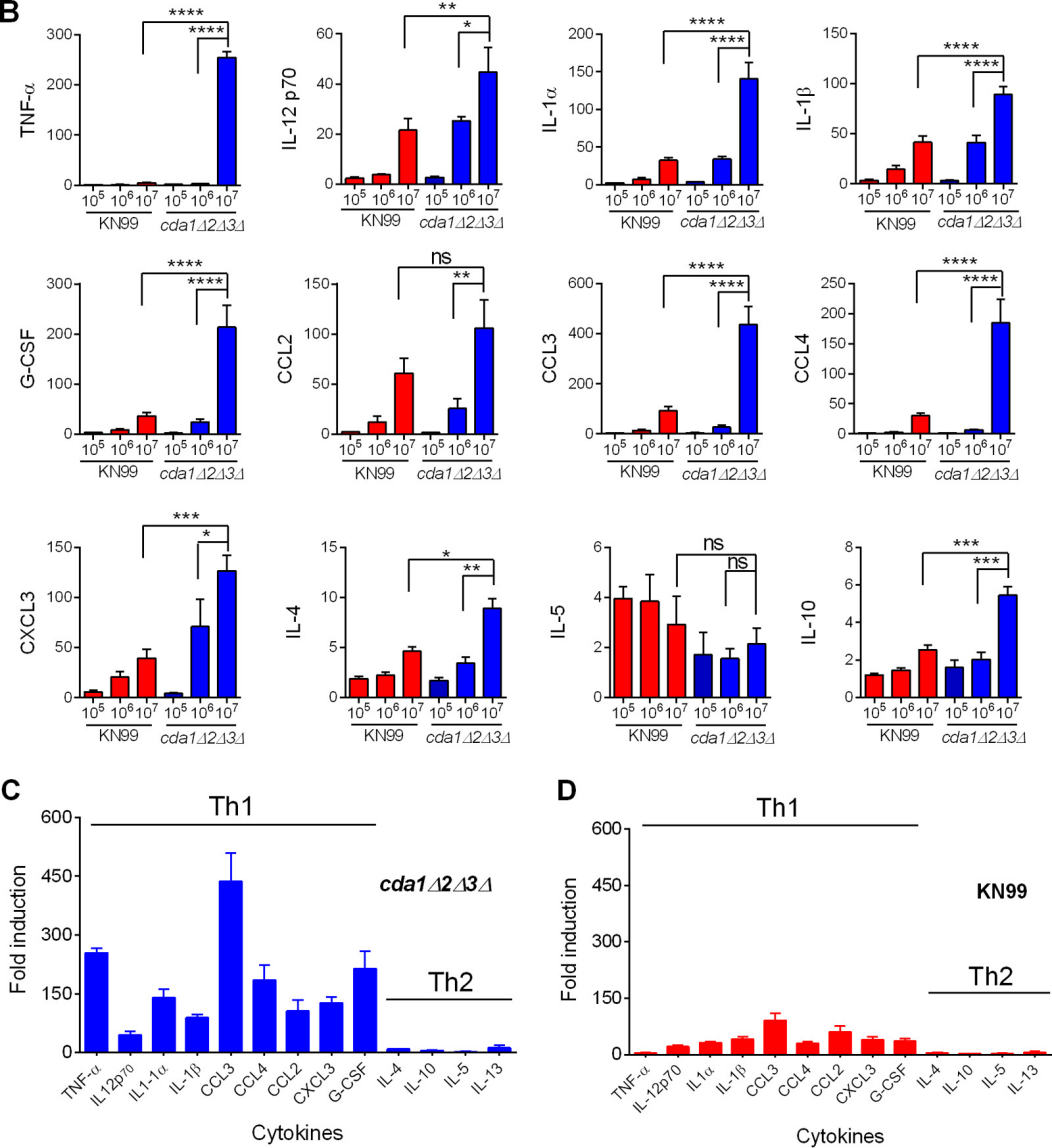
The kinetic profiles of mouse pulmonary cytokines were dependent on the inoculum load of *C. neoformans* cells and the presence or absence of chitosan in the cell wall. (A) Representative cytokine profiles in the lungs at various times after infecting the mice with various doses (equivalent to 10^5^, 10^6^, or 10^7^ CFU) of either KN99α or *cda1Δ2Δ3Δ* cells. Pulmonary cytokines that showed significantly different levels at day 3 p.i. in mice infected with 10^7^ CFU of KN99α or the *cda1Δ2Δ3Δ* mutant, in two independent experiments with three mice per treatment group, are displayed. Error bars indicate standard errors of the means. (B) Data from panel A are expressed as fold induction in protein levels of cytokines on day 3 p.i. in the lungs of the mice infected with various doses of either the *cda1Δ2Δ3Δ* mutant or KN99α compared to their levels in the PBS-inoculated control mouse lungs (*n* = 3). ****, *P* < 0.0001 by two-way ANOVA and Bonferroni’s multiple comparisons test. For IL2p70, **, *P* = 0.0053, and *, *P* = 0.0193; for CCL2, **, *P* = 0.002; for CXCL3, ***, *P* = 0.0006, and *, *P* = 0.026; for IL-4, **, *P* = 0.0027, and *, *P* = 0.05; and for IL-10, ***, *P* = 0.0009. All data are presented as mean values ± standard errors of the means (SEM). (C, D) Selective high-level induction of Th1-associated cytokines was observed only in *cda1Δ2Δ3Δ*-infected mouse lungs (C), while their upregulation was not as robust in KN99α-infected mouse lungs (D). The intensity of upregulation of Th2-associated cytokines was not as dramatic as that of the Th1 cytokines. The cytokine levels for each group were normalized to the results for the PBS control group.

To gain insight into the potential role of the host immune response in clearing the mutant fungal cells, we analyzed the concentrations of cytokines in lung homogenates at 1, 3, and 7 days p.i. by employing the Bio-Plex pro mouse cytokine assay (Bio-Rad Laboratories). We inoculated CBA/J mice with 10^5^, 10^6^, and 10^7^ CFU, as for the clearance studies. Overall, we found two major factors affecting the type and magnitude of the host cytokine response (Fig. 2; see also Table S1 in the supplemental material). First, the number of cells being introduced into the lung had a major influence on the magnitude of the host cytokine immune response: 10^5^ CFU gave minimal induction, while 10^7^ CFU elicited maximum induction. This trend was found for both the wild type and the *cda1Δ2Δ3Δ* strain (Fig. 2A). Second, the absence of chitosan in the cell wall of the *cda1Δ2Δ3Δ* mutant affected the timing and magnitude of the induction of various cytokines in the infected lungs. One of the characteristic features of the immune response triggered by *cda1Δ2Δ3Δ* mutant infection was that the levels of various cytokines peaked on day 3 p.i., before declining sharply on day 7 p.i. to around day 1 p.i. levels (Fig. 2A). However, in mice infected with wild-type KN99α, the concentrations of many cytokines gradually increased as the infection progressed (Fig. 2A).

We did not find statistically significant differences between the levels of cytokines in the lungs of mice infected with 10^5^ CFU of strain KN99α and the *cda1Δ2Δ3Δ* mutant (Fig. 2A and B; see also Table S1 in the supplemental material). However, with higher inocula, significant differences in the concentrations of cytokines were observed between KN99α- and *cda1Δ2Δ3Δ* mutant-infected lungs. With an inoculum of 10^6^ CFU, 14 cytokines (interleukin 1α [IL-1α], IL-1β, IL-12p70, tumor necrosis factor alpha [TNF-α], CCL3, CCL4, CCL2, IFN-γ, IL-2, IL-3, IL-4, IL-5, IL-10, and CCL5) showed significantly different levels in lung lysates of mice infected with KN99α and *cda1Δ2Δ3Δ* mutant cells on day 3 p.i. (see Table S1 in the supplemental material). When an inoculum of 10^7^ CFU was used, the levels of 11 of the 23 cytokines tested were significantly different in the lungs of mice infected with the wild type and the corresponding *cda1Δ2Δ3Δ* mutant on day 3 p.i. (Fig. 2A). Importantly, the magnitude of cytokine induction triggered by 10^7^ CFU of the *cda1Δ2Δ3Δ* mutant was distinct and dramatically higher than for all other infections (Fig. 2B).

We determined the fold induction of cytokines at day 3 p.i. in various *C. neoformans*-infected groups compared to the induction in the PBS-inoculated control group (Fig. 2B). Then, we compared the cytokine levels induced by 10^7^ CFU of the *cda1Δ2Δ3Δ* mutant to the levels induced by 10^7^ CFU of the wild type and 10^6^ CFU of the *cda1Δ2Δ3Δ* mutant. We consistently observed a larger induction of Th1-associated cytokines in mice infected with 10^7^ CFU of the *cda1Δ2Δ3Δ* mutant than in mice infected with the wild type or the lower inoculum of the *cda1Δ2Δ3Δ* strain. TNF-α, an important early-response cytokine, increased about 250-fold in the lungs of mice infected with 10^7^ CFU of the *cda1Δ2Δ3Δ* mutant, in contrast to 2- to 4-fold increases seen in mice infected with either 10^7^ CFU of KN99α or 10^6^ CFU of *cda1Δ2Δ3Δ* cells (Fig. 2B). Similarly, in mice infected with 10^7^ CFU of the *cda1Δ2Δ3Δ* mutant, cytokines IL-12, granulocyte colony-stimulating factor (G-CSF), IL-1α, and IL-1β increased about 45-, 215-, 140-, and 90-fold, respectively (Fig. 2B). IL-12p70 is an important proinflammatory cytokine that is known to promote the maturation of naive T cells to helper Th1 cells through the induction of IFN-γ (27). While both the KN99α and *cda1Δ2Δ3Δ* strains induced the upregulation of IL-12p70 in an inoculum size-dependent manner, its regulation was tightly controlled during infection with the *cda1Δ2Δ3Δ* mutant compared to its persistently higher levels in the KN99α-infected murine lung in the later days of infection (Fig. 2A). G-CSF specifically boosts the proliferation and maturation of neutrophils (28). The contribution of IL-1 in resisting fungal infections has been demonstrated using neutralizing antibodies to IL-1β and using IL-1 receptor-deficient (IL-1R^−/−^) mice (29).

Among the chemokines analyzed, macrophage inflammatory proteins CCL3 and CCL4, both of which are C-C chemokines, rose by 435- and 185-fold, respectively, in the lungs of mice infected with 10^7^ CFU of the *cda1Δ2Δ3Δ* mutant, while such a robust induction was not observed in the lungs of other mouse groups (infected with the wild type or a lower inoculum of the *cda1Δ2Δ3Δ* mutant) (Fig. 2B). We also saw statistically significant upregulation in the level of CXCL3, a CXCL-1–type chemokine that is important for attracting neutrophils to the site of infection, in the lungs of mice infected with 10^7^ CFU of the *cda1Δ2Δ3Δ* mutant (Fig. 2B). All the above-mentioned cytokines have been demonstrated to create an environment conducive for the development of a protective Th1-type adaptive immune response (30). In contrast, even though we observed statistically significant differences in the induction levels of IL-4 and IL-10 in mice infected with 10^7^ CFU of the *cda1Δ2Δ3Δ* mutant and mice infected with 10^7^ CFU of the wild type, the magnitudes of upregulation for infection with 10^7^ CFU of the *cda1Δ2Δ3Δ* mutant were small (5- to 10-fold) compared to the levels seen for the Th1-specific inflammatory molecules mentioned above (Fig. 2B and C). IL-4 and IL-10 are cytokines with known roles in the Th2-type immune response that are also known to facilitate fungal pathogenesis (31, 32).

Overall, we found that infection by and subsequent clearance of the *cda1Δ2Δ3Δ* strain triggered a host immune response characterized by significant, although temporally limited stimulation of cytokines known to be important for orchestrating a protective Th1-type environment in the lung (Fig. 2C). This Th1 cytokine enrichment in the lungs of mice infected with 10^7^ CFU of the *cda1Δ2Δ3Δ* mutant was in conjunction with lower levels of induction of the Th2-type cytokines IL-4, IL-5, IL-10, and IL-13. This suggested to us that the chitosan-deficient *cda1Δ2Δ3Δ C. neoformans* cells may be capable of skewing the host immune response in favor of a Th1-type adaptive response.

### Pulmonary inflammatory response triggered by infection with 10^7^ CFU of the *cda1Δ2Δ3Δ* mutant was followed by a specific increase in the recruitment of Th1 CD4^+^ T cells in the infected mouse lung.

**FIG 3:**
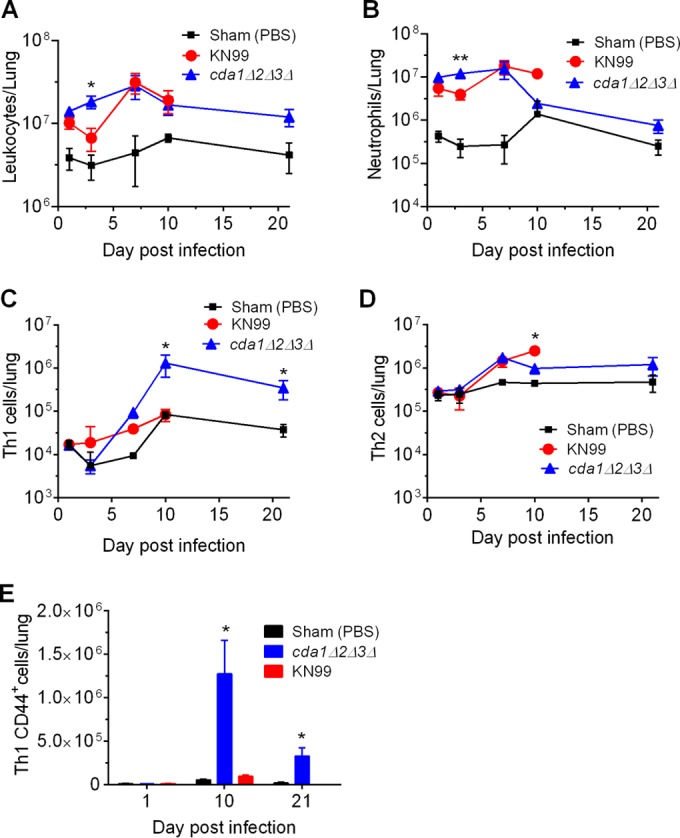
Pulmonary leukocyte analysis revealed increased recruitment of Th1-type CD4^+^ T cells in the lungs of mice infected with the chitosan-deficient *cda1Δ2Δ3Δ C. neoformans* cells. (A, B) Mice inoculated with 10^7^ CFU of the *cda1Δ2Δ3Δ* strain exhibited significant enrichment of total leukocytes (A) and neutrophils (B) in the lungs on day 3 p.i. compared to their levels in the lungs of mice inoculated with either 10^7^ CFU of KN99α or 50 µl of PBS. (C) The populations of CD4^+^ Th1 cells on days 10 and 21 p.i. were significantly higher in the *cda1Δ2Δ3Δ* mutant-infected mouse lungs than in the lungs of either PBS- or KN99α-inoculated animals. (D) The frequencies of Th2-type CD4^+^ leukocytes on day 10 p.i. were significantly higher in the KN99α-infected animals than in either PBS- or *cda1Δ2Δ3Δ*-inoculated animals. (E) Th1 cells induced during infection with *cda1Δ2Δ3Δ* cells showed increased expression of CD44 on their surface on day 10 p.i., and this was maintained during immune homeostasis (day 21 p.i.). The gating strategy for total leukocytes, neutrophils, Th1 and Th2 cells, and CD44 expression on Th1 cells per whole lung was based upon these leukocyte populations being CD45^+^, CD45^+^ CD24^+^ CD11c^−^ Siglec F-LY6G^+^, CD45^+^ CD3^+^ CD4^+^ CD183^+^ CD196^−^, CD45^+^ CD3^+^ CD4^+^ CD183^−^ CD196^−^, and CD45^+^ CD3^+^ CD4^+^ CD183^+^ CD196^−^ CD44^+^, respectively. The total number of leukocytes was calculated by multiplying the frequency of the leukocyte by the total number of cells determined from the single-cell mouse lung suspensions. Data are presented as the mean results ± standard deviations for three mice per group from one biological experiment. Means were compared among groups within each day using one-way ANOVA followed by the Bonferroni multiple-comparison test. *, *P* < 0.05; **, *P* < 0.01.

We conducted a pulmonary leukocyte analysis to study the recruitment of adaptive immune cells in response to infection. We infected mice with 10^7^ CFU of either KN99α or the *cda1Δ2Δ3Δ* mutant, and at days 1, 3, 7, 10, and 21 p.i., excised the lungs and prepared single-cell suspensions for analysis by multicolor flow cytometry. Since mice infected with KN99α did not survive beyond 19 days, they were not available for leukocyte analysis on day 21 p.i. The total number of leukocytes (i.e., CD45^+^) in the lungs was significantly higher for mice infected with the *cda1Δ2Δ3Δ* mutant than for either KN99α-infected or PBS control mice on day 3 p.i. (*P* < 0.05), which reached similar levels at the later stages of infection (Fig. 3A). However, the number of leukocytes in the KN99α-infected mice was not significantly higher than for the PBS control group on day 3. Similarly, there were significant increases in the numbers of neutrophils on days 1 (*P* = 0.0034) and 3 (*P* < 0.01) p.i. in the lungs of the mice infected with *cda1Δ2Δ3Δ* mutant compared to the numbers in KN99α-infected mice (Fig. 3B). However, no statistical differences were found between the total numbers of macrophages, dendritic cells, eosinophils, total CD4^+^ and CD8^+^ T cells, Th17 cells, and Treg cells for KN99α and *cda1Δ2Δ3Δ* mutant infection at each time point (data not shown). The frequencies of CD4^+^ T cells with a Th1 phenotype (CD45^+^ CD3^+^ CD4^+^ CD183^+^ CD196^−^) were significantly higher in the lungs of mice infected with *cda1Δ2Δ3Δ* cells (*P* < 0.05) than in KN99α-infected lungs on day 10 p.i. (Fig. 3C). In contrast, the number of CD4^+^ Th2 cells (CD45^+^ CD3^+^ CD4^+^ CD183^−^ CD196^−^) was significantly lower in *cda1Δ2Δ3Δ* mutant-infected lungs than in wild type-infected lungs (*P* < 0.05) on day 10 p.i. (Fig. 3D). Furthermore, significant populations of Th1 cells were found to express CD44 on day 10 p.i. only in the lungs of *cda1Δ2Δ3Δ* mutant-infected mice and not in the lungs of either KN99α-infected mice (*P* < 0.05) or PBS control mice (*P* = 0.0005) (Fig. 3E). High expression of CD44 receptor is associated with the expansion of Th1 cells into antigen-experienced memory cells (33). Taken together, the abundance of Th1-type T cells that were also CD44-expressing memory cells in the *cda1Δ2Δ3Δ* mutant-infected mouse lungs indicated that a host immune response triggered during the clearance of *cda1Δ2Δ3Δ* cells might induce protective immunity to subsequent *C. neoformans* infection.

### Immunizing mice with *cda1Δ2Δ3Δ* cells conferred complete protection to subsequent challenge with highly virulent wild-type *C. neoformans* cells.

**FIG 4:**
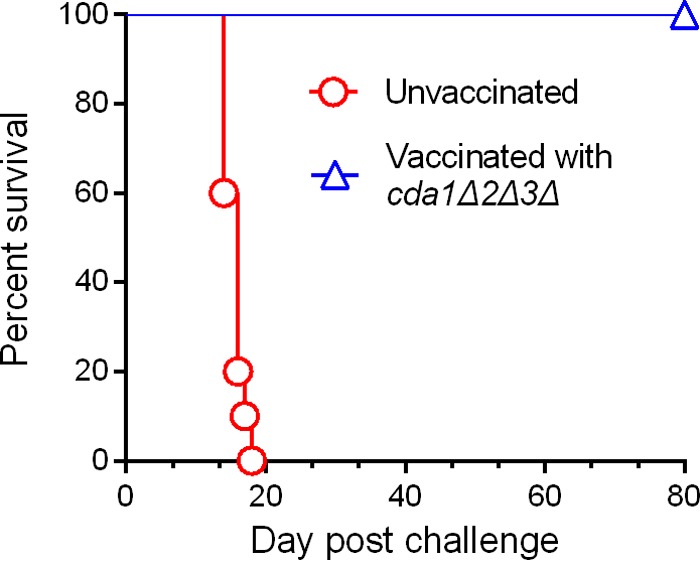
Vaccination of CBA/J mice with 10^7^ CFU of live *cda1Δ2Δ3Δ* cells conferred robust protective immunity to subsequent infection with wild-type KN99α *C. neoformans* cells. Mice were inoculated with 10^7^ CFU of *cda1Δ2Δ3Δ* cells through inhalation. PBS-inoculated mice served as control. Animals were left for 40 days to resolve the infection. Subsequently, both groups of mice were challenged with 10^5^ CFU of KN99α cells. Virulence was recorded as mortality of mice. Mice that lost 25% of starting body weight were considered to be moribund and were sacrificed. The percentage of mice that survived was plotted against the day p.i. Each survival curve is the average of three independent experiments that had five mice per experimental group.

To evaluate whether the pulmonary immune response elicited by the *cda1Δ2Δ3Δ* mutant and the subsequent increased recruitment of Th1-type CD4^+^ T cells is able to trigger protective immunity, we first infected CBA/J mice with 10^7^ CFU of *cda1Δ2Δ3Δ* cells in PBS through intranasal inoculation. Mice inoculated with PBS served as a control group. Mice were allowed 40 days to resolve the infection and then were challenged intranasally with 10^5^ CFU of the wild-type KN99α strain. Mice preinfected (immunized) with the *cda1Δ2Δ3Δ* strain were completely protected, while the PBS control group mice exhibited 100% mortality by day 19 postchallenge (Fig. 4). On day 80 p.i., the study was ended and the lung and brain homogenates of mice were plated for enumerating fungal CFU. We found that the fungal burden was restricted to the lung and was approximately equal to the challenge inoculum of 10^5^ CFU (data not shown).

### Heat-killed *cda1Δ2Δ3Δ* cells are equally effective in inducing strong protective immunity to *C. neoformans* infection.

**FIG 5:**
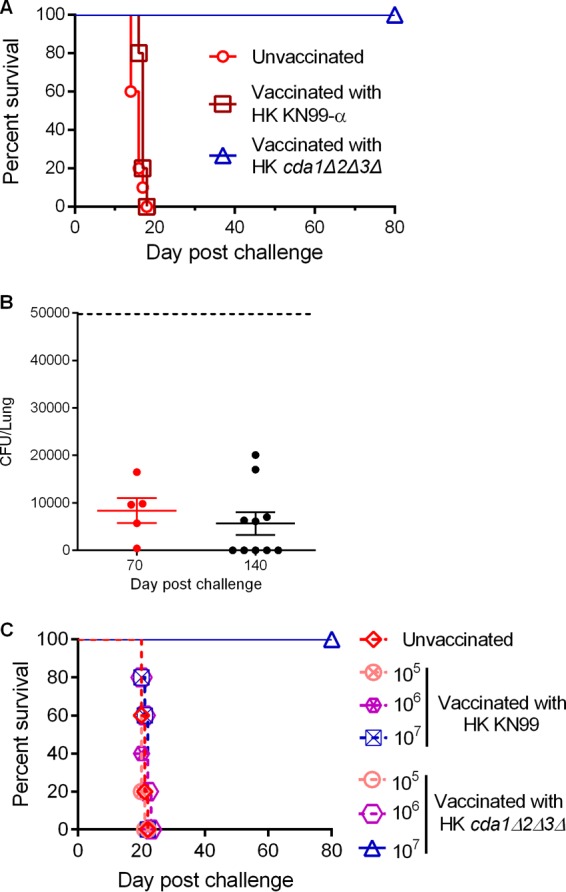
Heat-killed (HK) *cda1Δ2Δ3Δ* cells of *C. neoformans* induced strong protective immunity to a subsequent challenge with wild-type KN99α infection. (A) Mice were immunized with 10^7^ CFU of HK cells of either the wild-type KN99α or the *cda1Δ2Δ3Δ* strain. Control mice were inoculated with PBS. After 40 days, mice were challenged with 10^5^ CFU of wild-type KN99α cells. Survival of the animals was recorded as described above. The data shown are the average results from four experiments with five mice per experimental group. (B) Pulmonary fungal burden analysis of the mice that were vaccinated initially with 10^7^ HK *cda1Δ2Δ3Δ* cells and later challenged with 50,000 virulent KN99α cells. At 70 days p.i., lungs were harvested and fungal CFU were enumerated. (C) Mice were vaccinated with various doses (10^5^, 10^6^, or 10^7^ CFU) of HK cells of either the wild-type KN99α or the *cda1Δ2Δ3Δ* strain. After 40 days, they were challenged with 50,000 wild-type KN99α cells. Survival of the mice was monitored as described above.

To determine whether any biological activity present in the *cda1Δ2Δ3Δ* strain is specifically required for inducing protective immunity in mice, we wanted to test the feasibility of using its heat-killed (HK) cells as a vaccine. We determined that incubation of either KN99α or *cda1Δ2Δ3Δ* cells at 70°C for 15 min resulted in the complete loss of their viability, and we proceeded to inoculate mice intranasally with 10^7^ CFU of HK cells. At 40 days p.i., mice were challenged with 10^5^ CFU of the wild-type strain KN99α. We observed that mice inoculated with either PBS or HK KN99α succumbed to the KN99α challenge infection by day 19 (Fig. 5A). However, the mice vaccinated with HK *cda1Δ2Δ3Δ* cells were completely protected throughout the period of the experiment (80 days) (Fig. 5A). Similar to the results for vaccination with live *cda1Δ2Δ3Δ* cells, the fungal burden in the surviving mice was restricted to the lungs, with no dissemination to brain, spleen, or kidneys (data not shown). When 50,000 CFU of KN99α was used for challenging mice, we found on average an 85% decrease in the lung CFU on day 70 after the challenge infection, suggesting that HK *cda1Δ2Δ3Δ* mutant-induced protective immunity has the capacity to contain and partly clear an otherwise lethal infection. We also observed in a separate experiment that mice vaccinated with HK *cda1Δ2Δ3Δ* cells and later challenged with 50,000 CFU of KN99α cells survived for 140 days postchallenge, at which time the experiment was terminated (data not shown). Surviving mice from both these experiments showed various levels of fungal clearance, with some mice showing no fungal burden in the lung (Fig. 5B). In all mice, the fungal burden was restricted to the lungs. In the experiment that was ended on day 70, mice had a median fungal burden in the lungs of about 8,000 CFU/lung (Fig. 5B). The median fungal burden for the experiment that was ended on day 140 was around 5,500 CFU/lung (Fig. 5B).

Based upon our discovery that animals infected with 10^7^ CFU of *cda1Δ2Δ3Δ* cells responded with a vigorous induction of proinflammatory cytokines in the lungs, while the cytokine response was less dramatic in the mice infected with either 10^5^ or 10^6^ CFU, we wondered whether vaccination with lower doses (10^5^ or 10^6^ CFU) of heat-killed *cda1Δ2Δ3Δ* cells could induce protective immunity. To test this, we vaccinated mice with doses of 10^5^, 10^6^, and 10^7^ CFU of HK cells of either wild-type KN99α or the *cda1Δ2Δ3Δ* mutant. We found that mice vaccinated with 10^7^ HK *cda1Δ2Δ3Δ* cells were the only group that was able to mount a robust protective response to a subsequent lethal challenge with KN99α (Fig. 5C). Mice vaccinated with lower numbers of *cda1Δ2Δ3Δ* cells succumbed to infection similarly to those in the unvaccinated group, with a median survival time of between 20 and 22 days postchallenge (Fig. 5C). These data further support the requirement of a minimum dose of antigen to induce an effective early innate response for successful priming of an adaptive immune response.

### Vaccination of mice with the *C. neoformans cda1Δ2Δ3Δ* strain confers significant cross protection against subsequent pulmonary challenge with a lethal dose of *C. gattii.*

**FIG 6:**
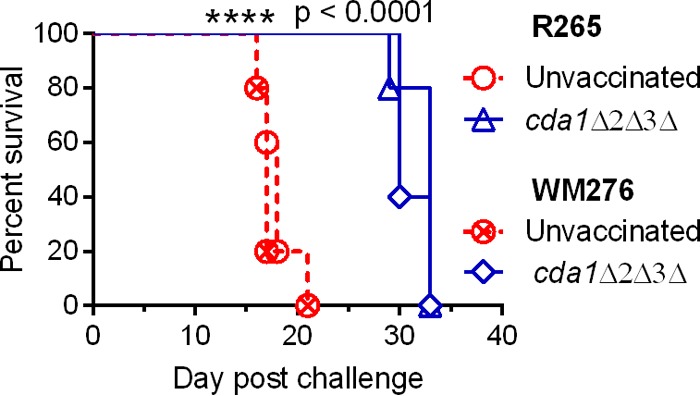
Vaccination of mice with HK cells of *cda1Δ2Δ3Δ C. neoformans* conferred partial protection to subsequent challenge with a lethal dose of a wild-type strain of *C. gattii*. A group of 10 mice (CBA/J) was subjected to initial vaccination with 10^7^ HK *cda1Δ2Δ3Δ* cells and later challenged with 10^5^ CFU of either *C. gattii* R265 or *C. gattii* WM276. Survival of the mice was monitored as described above. PBS-inoculated mice served as control.

Even though *C. neoformans* and *C. gattii* are closely related species, they differ significantly in their natural habitats, in the clinical manifestations of the disease they cause, in the specificity toward the organs they target in the murine infection model, in their carbon and nitrogen assimilation pathways, and finally, in the types of host immune response they provoke (34, 35). The differences in the immune responses they induce suggest that there may be differences in the organization of antigens on the cell surface of these two species. Therefore, we tested whether vaccination of mice with the *cda1Δ2Δ3Δ* mutant generated in a *C. neoformans* background would protect them from a subsequent challenge with *C. gattii*. Using the vaccination and infection protocol described above, we challenged mice vaccinated with HK cells of the *C. neoformans cda1Δ2Δ3Δ* mutant with 10^5^ CFU of either *C. gattii* strain R265 or *C. gattii* strain WM276. Mice vaccinated with PBS served as a sham vaccination control. We observed that control mice challenged with either *C. gattii* R265 or *C. gattii* WM276 had mean survival times of 18 and 17 days, respectively (Fig. 6). On the other hand, mice that were vaccinated with the *C. neoformans cda1Δ2Δ3Δ* strain exhibited significantly delayed mortality, with mean survival times of 33 and 30 days for *C. gattii* R265 and *C. gattii* WM276, respectively. This demonstrated that some cross protection developed with the *C. neoformans cda1Δ2Δ3Δ* vaccine.

### Heat-killed *cda1Δ2Δ3Δ* cell-induced protective immunity to *C. neoformans* infection is effective in multiple strains of mice.

**FIG 7:**
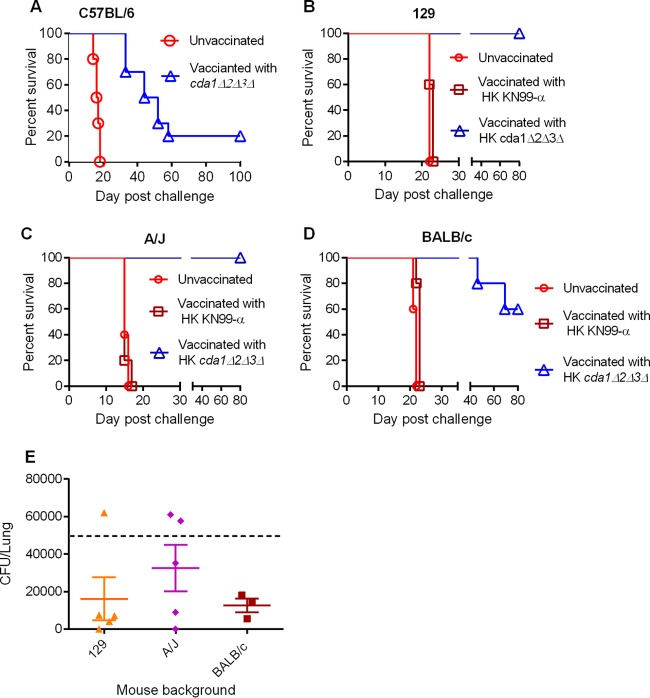
Protective immunity induced by HK *cda1Δ2Δ3Δ* cells was effective in different inbred mouse strains. (A) A group of 10 C57BL/6 mice were vaccinated with 10^7^ CFU of live *cda1Δ2Δ3Δ* cells and later challenged with 10^5^ CFU of KN99α cells as described above. Survival was monitored, and the percentage of survival was plotted against the day postchallenge. (B to D) Groups of five mice each of strains 129 (B), A/J (C), and BALB/c (D) were vaccinated with 10^7^ HK *cda1Δ2Δ3Δ* cells. At 40 days postvaccination, mice were challenged with 50,000 wild-type KN99α cells. Survival was monitored as described above. (E) At 80 days postchallenge, lungs from the surviving mice from the experiment whose results are shown in panels B, C, and D were harvested and subjected to lung fungal burden analysis.

Previous work by several laboratories demonstrated that mice of different genetic backgrounds exhibit various levels of sensitivity to cryptococcal infection (36–38). Therefore, we chose to test the effectiveness of the *cda1Δ2Δ3Δ* mutant vaccination in C57BL/6, 129, A/J, and BALB/c inbred mice. We vaccinated C57BL/6 mice with 10^7^ CFU of live *cda1Δ2Δ3Δ* cells and, after 40 days, challenged them with 10^5^ CFU of KN99α. Vaccination of C57BL/6 mice with the *cda1Δ2Δ3Δ* strain conferred a partial but significant level of protection, with a mean survival time of 48 days compared to 16.5 days in the unvaccinated control group (Fig. 7A).

We then vaccinated 129, A/J, and BALB/c mice with 10^7^ HK *cda1Δ2Δ3Δ* cells*.* The A/J and 129 mice were protected similarly to the CBA/J mice (Fig. 7B and C). All vaccinated animals survived the challenge with the KN99α strain for the length of the experiment (80 days postchallenge). However, for BALB/c mice, the efficacy of the *cda1Δ2Δ3Δ* mutant vaccine was slightly decreased (Fig. 7D). Of the five mice used for the experiment, three survived the infection, while two succumbed, on day 46 and day 64 (Fig. 7D). All mice either inoculated with PBS or vaccinated with an HK preparation of KN99α cells succumbed by 23 days postchallenge. Among these control groups, the A/J mice were the most sensitive to KN99α challenge, with a median survival time of 15 days, while 129 and BALB/c mice showed median survival times of 22 and 23 days, respectively. We also observed significant but varied clearance of infection in vaccinated mice of different genetic backgrounds (Fig. 7E).

## DISCUSSION

Our initial goal for the investigation was to understand the mechanism of the clearance of chitosan-deficient mutant strains of *C. neoformans* in the mouse lung. In our previous study, we reported that cells of *C. neoformans chs3*Δ, *csr2*Δ, and *cda1Δ2Δ3Δ* mutant strains were devoid of chitosan and displayed compromised cell wall integrity (26). Interestingly, while the *chs3Δ* and *csr2Δ* strains were temperature sensitive, the *cda1Δ2Δ3Δ* strain was able to grow at host body temperature. Therefore, we selected the *cda1Δ2Δ3Δ* strain to further understand the mechanism of its clearance from the mouse lung. Since 10^5^ CFU of the *cda1Δ2Δ3Δ* mutant were cleared in 24 h, we wondered whether increasing the CFU of the inoculum would alter clearance (25). We found that using higher inocula delayed complete eradication (Fig. 1). Moreover, when 10^7^ CFU of the *cda1Δ2Δ3Δ* mutant were used, we did recover 5,000 CFU of cells from the infected lung at 7 days p.i., showing that *cda1Δ2Δ3Δ* cells were able to survive in the host. It will be interesting to see whether those that survived for 7 days were the product of newly replicated fungal cells or persisted from the original inoculum without replication. Nevertheless, the complete clearance of *cda1Δ2Δ3Δ* cells from the mouse lung by day 10 p.i. suggested that host-specific mechanisms may be responsible for clearing the chitosan-deficient mutant cells, as opposed to the mere inability of the *cda1Δ2Δ3Δ* cells to survive or grow in the host lung.

The results from using increasing sizes of *cda1Δ2Δ3Δ* inocula for intranasal inoculation not only suggested the potential role of host factors in eradicating the mutant fungal cells but also implied that the intensity of the host immune response was dependent on the size of the initial infection. We chose to measure cytokine levels in lung homogenates as a measure of the host response. Infection with 10^5^ CFU resulted in the detection of low levels of cytokines on different days p.i. This may be due to either the limitations in the sensitivity of the assay employed or the difficulty in choosing an ideal time point for cytokine analysis due to the dynamic nature of the inflammatory response induced. Moreover, at this size of inoculum, we did not find a statistically significant difference between the levels of cytokines in KN99α- and *cda1Δ2Δ3Δ* mutant-infected murine lungs. However, there were statistically significant differences between the amounts of various cytokines in the lung homogenates of mice infected with KN99α and with the *cda1Δ2Δ3Δ* mutant for mice inoculated with both 10^6^ and 10^7^ CFU, and the magnitude of their induction on day 3 p.i. was clearly different for the mice infected with 10^7^ CFU.

TNF-α has been shown to be necessary for the development of Th1-mediated immunity and plays a pivotal role in clearing various fungal infections, including *C. neoformans* infection, in animal models (reference 39 and references therein). The production of TNF-α in the afferent phase (0 to 7 days p.i.) of the host immune response has been demonstrated to be critical for the development of protective immunity to *C. neoformans* infection in the CBA/J mouse model (40). Such a dependency of *cda1Δ2Δ3Δ* mutant-induced protective immunity on the early expression of TNF-α may be playing a role in our experiments. While infection with 10^5^ and 10^6^ CFU of *cda1Δ2Δ3Δ* cells did not elicit significant TNF-α induction, infection with 10^7^ CFU of the *cda1Δ2Δ3Δ* mutant clearly produced a dramatic spike in its levels in the lung on day 3 p.i. Accordingly, vaccination with 10^5^ and 10^6^ CFU of the *cda1Δ2Δ3Δ* mutant did not confer any protection. However, robust protection was observed when 10^7^ CFU of the *cda1Δ2Δ3Δ* mutant was used for vaccination. Therefore, vaccination experiments when performed in the presence of TNF-α-specific neutralizing antibodies may shed light on the essential nature of early TNF-α induction during vaccination, on the clearance of the *cda1Δ2Δ3Δ* strain, and on subsequent induction of effective protective immunity to *C. neoformans* infection. More importantly, the increased levels of TNF-α detected during infection with 10^7^ *cda1Δ2Δ3Δ* cells did not persist after 3 days p.i. This might perhaps have prevented deleterious damage to the host lung due to an unregulated TNF-α-mediated inflammatory response. Several fungus-related virulence factors, such as polysaccharide capsule, melanin, and prostaglandinlike molecules, have been documented to inhibit the stimulation of TNF-α during wild-type *C. neoformans* infection (41–43). However, determination of cellular factors associated with the *cda1Δ2Δ3Δ* strain that resulted in the controlled expression of TNF-α production during infection will provide more insight into the regulation of TNF-α during *C. neoformans* infection. In any case, this effect of the *cda1Δ2Δ3Δ* strain in controlled regulation of TNF-α expression during vaccination may be an added benefit as far as the safety of the vaccine is concerned.

IL-12 is a heterodimeric cytokine that has been shown to be essential for the development of a protective Th1 response and resistance to *C. neoformans* infection, and its expression is under the influence of TNF-α (39, 44, 45). We found that the expression of IL-12p70 in both KN99α- and *cda1Δ2Δ3Δ* mutant-infected lungs followed the pattern of TNF-α expression (see Table S1 in the supplemental material). We did not detect significant differences between the levels of either IFN-γ or IL-2 in KN99α- and *cda1Δ2Δ3Δ* mutant-infected mouse lungs at the chosen time points. This may be due to the complex and dynamic nature of the regulation of cytokine expression, the limited number of time points chosen in our experiment for cytokine analysis, or the limitations in the sensitivity of the assay employed.

IL-1α and IL-1β both belong to the IL-1 family of proinflammatory cytokines that play a major role in the host’s defense against infections, not only by activating innate immune cells but also by triggering further release of proinflammatory cytokines that participate in the modulation of the host adaptive immune response. The critical role of IL-1β in controlling *C. neoformans* and other pathogenic fungal infections through the activation of the NLRP3 inflammasome has recently been documented (46–48). We do not know the source of IL-1β in the present study; however, dendritic cell-derived IL-1β has been shown to be important for effective priming of T cells (49). Moreover, when recombinant IL-β was used as an adjuvant together with a heat-killed formulation of a weak attenuated strain of *Blastomyces dermatitidis*, it enhanced the protective effect of the vaccine strain (50). Even though infecting mice with 10^7^ CFU of KN99α caused the stimulation of both IL-1α and IL-1β, the levels were only comparable to those induced by infection with 10^6^ CFU of the *cda1Δ2Δ3Δ* mutant, which was nonprotective. Therefore, the timing (3 days p.i.) and the effective concentration of both IL-1α and IL-1β induced by 10^7^ CFU of the *cda1Δ2Δ3Δ* mutant may have contributed to the protective immunity.

CCL3 is a C-C chemokine that acts as a chemoattractant for leukocytes, is critical for the promotion of the Th1-type immune response, inhibits the expression of IL-4/IL-13, and plays an important role in preventing lung eosinophilia during *C. neoformans* infection (51, 52). Of all the cytokines tested, CCL3 showed a maximum intensity of induction in the *cda1Δ2Δ3Δ* mutant-infected mouse lung which was specific to mice infected with 10^7^ CFU of cells, similar to the response seen for TNF-α. Consistent with this, we saw a significant increase in leukocyte migration to the lungs on day 3 p.i. in the 10^7^ *cda1Δ2Δ3Δ* mutant-infected mouse lung compared to that in the KN99α-infected mouse lung. Therefore, CCL3 may be one of the important cytokines responsible for the migration of leukocytes to the lung. These results, along with the previously documented requirement of early induction of CCL3 during *C. neoformans* infection in promoting the Th1-type protective cellular response, suggest a role of CCL3 in the *cda1Δ2Δ3Δ* mutant-induced protective immunity (52).

CCL2 is a potent chemotactic factor for monocytes and dendritic cells, in addition to its role in regulating the migration and infiltration of memory T-lymphocytes and natural killer (NK) cells. The role of CCL2 in inducing Th1 protective immunity and fungal clearance during *C. neoformans* infection has been demonstrated by its involvement in recruiting macrophages, CD4^+^ T cells, and NK T cells to the site of infection (53, 54). Even though we observed increased levels of CCL2 in the lungs of mice infected with 10^7^ CFU of the *cda1Δ2Δ3Δ* mutant, significantly increased levels of CCL2 were also seen in mice infected with 10^7^ CFU of strain KN99α. These results suggest a minimal role of CCL2 in *cda1Δ2Δ3Δ* mutant-induced protective immunity.

CXCL3 is a CXCL1 type of chemokine that is associated with neutrophil recruitment. The contribution of CXCL3 in inducing resistance to *C. neoformans* infection is not well characterized. However, we observed that this chemokine was significantly elevated in murine lungs infected with 10^7^ CFU of the *cda1Δ2Δ3Δ* mutant. This is consistent with previously published results showing the expression pattern of CXCL3 during infection of the SJL/J mouse strain that is resistant to a moderately virulent strain of *C. neoformans* (strain 24067) (55). The higher concentration of CXCL3, followed by the increased number of neutrophils in the lungs of mice infected with 10^7^ CFU of the *cda1Δ2Δ3Δ* mutant, may indicate an important role of this chemokine in the clearance of the *cda1Δ2Δ3Δ* mutant and in the induction of adaptive immunity. In addition, the significant increase in the levels of G-CSF, only in the lungs of mice infected with 10^7^ CFU of the *cda1Δ2Δ3Δ* mutant, might have promoted the proliferation and maturation of neutrophils, which is consistent with previously published results showing a beneficial role of recombinant G-CSF therapy during bacterial and opportunistic fungal infections (28). Furthermore, recombinant human G-CSF has been reported to enhance the ability of human effector cells to kill *C. neoformans* (56). Therefore, we speculate that this property of G-CSF may be conserved and may be partly responsible for the clearance of the *cda1Δ2Δ3Δ* mutant. Cytokines secreted upon the infiltration of potent neutrophils might have contributed to the eventual polarization of the adaptive immunity and the subsequent protection against wild-type *C. neoformans* challenge.

The selective induction of the above-mentioned proinflammatory cytokines during infection with 10^7^ CFU of the *cda1Δ2Δ3Δ* mutant was associated with minimal changes in the concentrations of cytokines driving a Th2 response, such as IL-4, IL-5, IL-10, and IL-13 (Fig. 2C). A Th2-type T cell response exacerbates *C. neoformans* infection by allowing fungal proliferation (31, 57). Overall, our cytokine analysis suggested an overwhelming proinflammatory response that should direct Th1 skewing of the early host immune response. This was supported by the results of multicolor flow cytometry, which showed an increase of Th1-type cells in the infected lung on day 10 p.i. This immunological data prompted us to evaluate whether chitosan-deficient *cda1Δ2Δ3Δ* cells can induce protective immunity.

Unlike *C. neoformans*, *C. gattii* causes disseminated infections in immunocompetent and apparently healthy individuals. *C. gattii* strain R265, which was responsible for the Pacific Northwest outbreak, was found to be more virulent than *C. gattii* strain WM276, an environmental isolate from Australia, when tested for virulence in either C57BL/6 or A/JCr mice (58). Interestingly, we observed similar levels of virulence for these strains in the CBA/J mouse model both in the immunized and naive mouse groups. It is encouraging that the *cda1Δ2Δ3Δ* mutant engineered in *C. neoformans* generated significant cross protection to *C. gattii* infections. This is most likely due to the presence of common immunodominant proteins in the cell wall fraction of both *C. neoformans* and *C. gattii* (30, 59). However, the failure of the *C. neoformans cda1Δ2Δ3Δ* vaccine to confer complete protection against *C. gattii* infection emphasizes the underlying differences between the antigenic determinants in these two species of *Cryptococcus*. In addition, previous studies have reported that *C. gattii* infection causes inhibition of efficient neutrophil migration and pulmonary cytokine production and downregulation of the dendritic cell-mediated Th1 immune response (34, 58). Therefore, it is plausible that challenging *cda1Δ2Δ3Δ* strain-vaccinated mice with *C. gattii* might have decreased the intensity of the protective host inflammatory response, which in turn resulted in the less-effective cross protection observed in our experiments. The mechanisms of chitin and chitosan biosynthesis and its regulation in *C. gattii* have not been studied. It will be interesting to see whether vaccination with a *cda1Δ2Δ3Δ* strain engineered in *C. gattii* confers complete protection against *C. gattii* infections.

Several studies have demonstrated the influence of host genetic factors on the susceptibility to *Cryptococcus* infection (36, 37). CBA/J mice have been demonstrated to be resistant to *C. neoformans* strain 52 D by developing a strong Th1 adaptive immunity that results in the progressive clearance of the infection. In contrast, C57BL/6 mice respond with a skewed Th2 response and succumb to infection. In such infection experiments, BALB/c mice have exhibited an intermediate phenotype (57). These patterns are mirrored by the results of our vaccination and challenge experiments. CBA/J mice showed a strong resistance to infection after vaccination, with significant clearing, while the efficiency of protection was slightly reduced in BALB/c mice and appreciably decreased in C57BL/6 mice. Interestingly, all the surviving BALB/c mice showed significant clearance of the challenge inoculum. The vaccination of 129 and A/J strains with the *cda1Δ2Δ3Δ* mutant resulted in the generation of protective immunity similar to that in the CBA/J strain, with all mice showing clearance of the challenge inoculum to various degrees. Even though naive A/J mice were more sensitive to *C. neoformans* infection than 129 and BALB/c mice, they were able to mount a strong protection after vaccination.

Previous reports have shown that commercial and fungal chitin preparations elicit potent immune responses in the host (60–62). The type of immune response stimulated by a chitin preparation depended on its source, size, purity, and concentration in *in vitro* and *in vivo* studies (63, 64). Purified chitosan also stimulates immune responses, including inflammasome activation, which suggests a proinflammatory role for chitosan as well (65). However, the biological context of the material being presented may be crucial for how it is interpreted by the host. Additional pathogen-associated molecular patterns (PAMPs) present in the wild-type cell surface may be modulating a host response to whole *Cryptococcus* cells that is distinct from the response to purified chitosan.

We have previously shown that all of the chitin-derived fiber in the *cda1Δ2Δ3Δ* mutant and most of the chitin-derived material in *chs3Δ* and *csr2Δ* cells exists in its fully acetylated chitin form. These chitosan-deficient mutants exhibited sensitivity to cell wall stressors and a leaky melanin phenotype, suggesting a potentially disorganized cell wall architecture with possible exposure of underlying chitin and other immunogenic molecules on the cell surface which, in turn, may have resulted in the induction of a strong host immune response (26). It is possible that the chitin induces the protective immune response. The nature of the chitin fiber present in the *cda1Δ2Δ3Δ*, *chs3Δ*, and *csr2Δ* strains is not known, but all of the strains were cleared rapidly from the infected murine lung (25). In the case of the *chs3Δ* and *csr2Δ* strains, their clearance can almost surely be attributed in part to their temperature-sensitive phenotype. However, whether their clearance is followed by a concomitant polarization of the host immune response toward Th1 adaptive immunity is yet to be determined, which may provide more insight into the connection between chitin/cell wall structure and the host immune response. In wild-type cells, the presence of chitosan may mask the exposure of the chitin, resulting in a subdued host inflammatory response. It is possible that the host response is due to the exposure of chitin and not the quantity of chitin in the cell wall.

Various *C. neoformans* cell wall-associated proteins have been shown to induce a proinflammatory response that was followed by partial protection against *C. neoformans* infection when they were used as vaccines by themselves or in conjunction with yeast glucan particles (18, 59). Lack of chitosan in the *cda1Δ2Δ3Δ* strain may have resulted in the exposure of these antigenic cell wall proteins and other potential PAMPs which, in addition to the potent immunomodulatory property of chitin, may have contributed to the mechanisms of protective immunity. Comparison of the surface-exposed PAMPs on additional chitin or chitosan mutants or mutants with other mutations that affect the cell surface, such as Znf2 overexpression (which causes a morphogenesis defect) and Rim101 deletion (which causes cell wall defects), and the nature of the host immune responses may lead to a better understanding of the molecular signals that drive the protective response (24, 66).

In the absence of potent subunit vaccines that confer complete protection, the use of attenuated strains as potential vaccine candidates is one of the most promising approaches. The finding that HK cells of the *cda1Δ2Δ3Δ* mutant conferred protective immunity makes it attractive for development into a safe vaccine for clinical use.

## MATERIALS AND METHODS

### Mice.

All mice used were females between 6 and 8 weeks old. CBA/J, C57BL/6, 129, BALB/c, and A/J mice were from The Jackson Laboratory (Bar Harbor, ME). Mice were housed at the Washington University School of Medicine and handled according to guidelines approved by the Institutional Animal Care and Use Committee.

### Cryptococcus strains.

*C. neoformans* strain KN99α and the *cda1Δ2Δ3Δ* (LBCN 632) strain derived from it were described previously (25). *C. gattii* strain R265 is a representative of the common VGIIa molecular subtype and is from the outbreak in British Columbia. *C. gattii* strain WM276 is a representative of subtype VGI and is an environmental isolate from Australia. Both of these strains were kindly provided by Joseph Heitman (Duke University Medical Center). Cells were recovered from −80°C stocks prior to use in each experiment. The strains were cultured in yeast extract-peptone-dextrose (YPD) medium*.*

### Pulmonary infections.

Yeast strains were grown in 50 ml of YPD medium in a 250-ml Erlenmeyer flask (Corning Incorporated, Tewkesbury, MA) for 36 h at 30°C and at 300 rpm in a shaking incubator. Cells were harvested by centrifugation at 1,800 × *g* for 10 min, washed twice with phosphate-buffered saline (PBS) and resuspended in PBS at an appropriate cell density. Mice were infected intranasally as previously described (25). Briefly anesthetized mice were suspended by their incisors on a thread. A yeast inoculum of 50 µl containing the appropriate number of yeast cells was slowly dripped directly into the nares. Mice were left suspended for an additional 10 min and were closely monitored until they recovered from anesthesia. Mice were monitored daily and those showing signs of morbidity (weight loss or extension of the cerebral portion of the cranium) were sacrificed by CO_2_ asphyxiation.

### Pulmonary fungal burden and cytokine analysis.

At various time points after infection, mouse organs were excised, weighed, and homogenized in 2 ml of PBS containing Complete protease inhibitor cocktail tablet (Roche Diagnostics) using a Pro200 homogenizer (Pro Scientific, Oxford, CT). Homogenates were serially diluted, plated on YPD agar, and incubated at 30°C. Yeast colonies were counted after 2 to 3 days of incubation. For pulmonary cytokine analysis, 0.05% Triton X-100 was added to a portion of the homogenized lung lysate and the sample was incubated for 2 h at 4°C with intermittent mixing. At the end of incubation, the lysate was centrifuged at 12,000 × *g* for 20 min. The supernatant was collected and spun again for 20 min at 12,000 × *g*. Aliquots of the supernatant were stored at −80°C. Cytokine analysis was performed using the Bio-Plex pro mouse cytokine 23-plex assay kit, following the protocol supplied with the kit (Bio-Rad Laboratories, Hercules, CA). Fifty microliters of lung sample was analyzed in each well.

### Preparation of lung leukocyte single-cell suspensions.

Mice were sacrificed, and their left lungs were removed and added to homogenization C tubes containing proprietary catalysts for mechanical and enzymatic digestion (Miltenyi Biotec, Auburn, CA). This was followed by homogenization of lung tissue in the gentleMACS dissociator based upon the manufacturer’s instructions (Miltenyi Biotec). Homogenized tissue was then passed through the 70-µm-pore-size MACS SmartStrainer (Miltenyi Biotec) and centrifuged at 300 × *g* for 10 min to pellet cells. Erythrocytes were removed using ACK (ammonium-chloride-potassium) lysing buffer (Gibco, Grand Island, NY). Briefly, 1 ml of ACK buffer was used to suspend pelleted cells. After a maximum of 10 min, the ACK lysing buffer was diluted 1:10 with complete RPMI medium. Cells were then washed with cell staining buffer (CSB) containing final concentrations of 1 × PBS (Corning Cellgro, Manassas, VA), 2 mM EDTA (Amresco, Solon, OH), and fraction V BSA (Fisher Scientific, Pittsburgh, PA). Cells were then counted in a hemocytometer, using trypan blue staining to exclude dead cells.

### Fluorochrome-conjugated antibodies for flow cytometry.

The following fluorochrome-conjugated mouse antibodies from BioLegend (San Diego, CA) were used: peridinin chlorophyll protein (PerCP)-Cy5.5-conjugated anti-CD45 antibody (Ab) (30-F11), phycoerythrin (PE)-conjugated anti-CD24 Ab (30-F1), allophycocyanin (APC)-conjugated anti-CD11c Ab (N418), PE-Cy7-conjugated anti-CD206 Ab (C068C2), and PE-Cy7-conjugated anti-CD Ly-6G Ab (1A8). The following BD Biosciences (San Jose, CA) fluorochrome-conjugated antibodies were also used: BUV737-conjugated anti-CD11b Ab (M1/70), BV605-conjugated anti-major histocompatibility complex class II (MHC-II) (M5/114.15.2), BV421-conjugated anti-CD80 Ab (16-10A1), Alexa Fluor 647-conjugated anti-Siglec F Ab (E50-2440), BV711-conjugated anti-CD3e Ab (145-2C11), Alexa Fluor 700-conjugated anti-CD4 Ab (RM4-5), BB515-conjugated anti-CD25 Ab (PC61), APC-H7-conjugated anti-CD8a Ab (53-6.7), BV605-conjugated anti-CD27 Ab (LG.3A10), PE-CF594-conjugated anti-CD28 Ab (37.51), BV421-conjugated anti-CD183 Ab (CXCR3-173), Alexa Fluor 647-conjugated anti-CD196 Ab (140706), BUV737-conjugated anti-CD44 Ab (IM7), and PE-conjugated anti-CD45RB Ab (16A).

### Flow cytometry analysis of leukocyte populations.

Briefly, cell surface immunofluorescence staining involved the addition of 0.015 to 0.15 µg of a fluorochrome-conjugated antibody mixture containing antibodies specific to various leukocyte subpopulations to 25 µl of an equal mixture of CSB (defined above) and BD Horizon Brilliant stain buffer (San Jose, CA) in the wells of a 96-well microtiter plate. This staining mixture was then used to resuspend 2.5 × 10^5^ cells in triplicate. Cells were then incubated on ice for 30 min in the dark. This was followed by two washes with CSB, and then cells were fixed at 4°C overnight in 1% paraformaldehyde (PFA). The next day, cells were washed and resuspended in CSB and added to a 96-well microtiter plate for high-throughput flow cytometry sampling using a BD LSRFortessa X-20 (BD Biosciences). BD FACSDiva software was used to process samples on the flow cytometer, and FlowJo (FlowJo, LLC, Ashland, OR) was used for data analysis of the acquired samples to facilitate the identification of leukocyte populations. Thirty thousand cell events were recorded, and dead cells and debris were excluded based upon lower forward scatter (FSC) and side scatter (SSC) signals (i.e., smaller size and granularity). Leukocyte populations were identified by the following markers as previously described: eosinophils (CD45^+^ CD24^+^ Siglec F^+^), neutrophils (CD45^+^ CD24^+^ LY6G^+^), CD11b^+^ dendritic cells (DCs) (CD45^+^ CD11b^+^ CD11c^+^ CD24^+^ MHC-II^+^), CD11b^−^ DCs (CD45^+^ CD11b^−^ CD11c^+^ CD24^+^ MHC-II^+^), alveolar (CD45^+^ CD11b^−^ CD11c^+^ CD24^−^ MHC-II^+^) and interstitial (CD45^+^ CD11b^+^ CD11c^+^ CD24^−^ MHC-II^+^) macrophages, Th1 CD4 T cells (CD45^+^ CD3^+^ CD4^+^ CD183^+^ CD196^−^), Th2 CD4 T cells (CD45^+^ CD3^+^ CD4^+^ CD183^−^ CD196^−^), Th17 cells (CD45^+^ CD3^+^ CD4^+^ CD183^−^ CD196^+^), regulatory T cells (Tregs) (CD45^+^ CD3^+^ CD4^+^ CD25^+^), CD8 cytotoxic T cells (CD45^+^ CD3^+^ CD8^+^ CD28^+^), and CD8 T suppressor cells (CD45^+^ CD3^+^ CD8^+^ CD28^−^) (34, 67, 68). The absolute number of each leukocyte population was determined by multiplying the frequency of the leukocyte by the number of cells determined from the single-cell suspensions and then multiplying by 2 to account for the whole lung.

### Statistical analysis.

All statistical analyses were performed using GraphPad Prism version 6.0 (GraphPad Software, Inc., San Diego, CA). Statistical significance for survival studies was assessed by log-rank test. Other group comparisons were performed by two-way analysis of variance (ANOVA) with Bonferroni’s multiple-comparison test.

## SUPPLEMENTAL MATERIAL

Table S1  Kinetic profiles of various cytokines in the lungs of the mice infected with various doses (equivalent to CFU of 10^5^ or 10^6^ or 10^7^) of either the wild-type KN99α or the *cda1Δ2Δ3Δ* mutant strain, determined at 1, 3, and 7 days postinfection. Lungs were excised and homogenized as described in Materials and Methods, and 50 µl of the homogenate was used for the assay. Concentrations of the cytokines are expressed as pg/ml of the homogenate. Comparisons of the concentrations of the various cytokines were performed between the lungs of the mice infected with KN99α and with the corresponding *cda1Δ2Δ3Δ* strain. Data for the 10^7^-CFU infection are representative of two biological experiments with three mice per each time point. Data for the 10^5^- and 10^6^-CFU infections are from one biological experiment with three animals per each time point. *, *P* < 0.05 compared to the corresponding wild-type or *cda1Δ2Δ3Δ* mutant strain-infected lung homogenate. Table S1, XLSX file, 0.02 MB
